# An integrated analysis reveals the oncogenic function of lncRNA LINC00511 in human ovarian cancer

**DOI:** 10.1002/cam4.2171

**Published:** 2019-04-23

**Authors:** Jing Wang, Yongju Tian, Hui Zheng, Yan Ding, Xiuli Wang

**Affiliations:** ^1^ Department of Obstetrics and Gynecology The Affiliated Yantai Yuhuangding Hospital of Qingdao University Yantai, Shandong P.R China; ^2^ Department of Gynecology Yantaishan Hospital Yantai, Shandong P.R China; ^3^ Department of Gastrointestinal Surgery The Affiliated Yantai Yuhuangding Hospital of Qingdao University Yantai, Shandong P.R China; ^4^ Department of Spine Yantaishan Hospital Yantai, Shandong P.R China

**Keywords:** EZH2, LINC00511, long noncoding RNA, ovarian cancer, P21

## Abstract

Ovarian cancer is one of the most common female reproductive system malignancies worldwide. Recently, the aberrant long noncoding RNAs (lncRNAs) expression has been identified in multiple cancers. Emerging evidence has highlighted the critical roles of lncRNAs in carcinogenesis and tumor progression, including ovarian cancer. The objective of this study is to comprehensively analyze lncRNAs expression pattern, and explore their clinical significance and underlying mechanism in human ovarian cancer. In this study, we found hundreds of dysregulated lncRNAs in ovarian cancer by performing genome‐wide analysis using RNA sequencing data from Genotype‐Tissue Expression (GTEx) and The Cancer Genome Atlas (TCGA) project, and three microarray datasets from Gene Expression Omnibus (GEO). Moreover, our results revealed that up‐ or down‐regulation of some lncRNAs expression in ovarian cancer is accompanied by their genomic loci copy number amplification or deletion. Importantly, some lncRNAs expression levels are significantly associated with ovarian cancer patients’ poor prognosis. Further experimental validation and mechanistic investigation indicate that LINC00511 exerts oncogenic function in ovarian cancer cells through interacting with EZH2 and repressing P21 expression. Taken together, the findings in the current study may provide a useful resource of novel ovarian cancer associated lncRNAs and potential diagnostic biomarker and therapeutic targets for ovarian cancer.

## 1 INTRODUCTION

Ovarian carcinoma is one of the most common reproductive system malignancies in female residents, and has been one of the leading causes of cancer‐related death worldwide.^1^ In spite of the great advancements in surgical technique, chemotherapeutic treatment, and targeted chemotherapy, the prognosis of ovarian cancer patients remains unsatisfactory, with an ~30% 5 years overall survival rate.^2^, ^3^ In addition, patients with ovarian cancer often had an poor outcome due to the rapid tumor progression, occurrence of cancer cells metastasis, and limited therapeutic strategies.^4^, ^5^ Hence, a better understanding of the pathophysiological process and underlying mechanisms contributing to ovarian cancer development and progression is of paramount importance to identify novel biomarkers and targets for ovarian cancer diagnosis and therapy.

In recent years, a growing number of evidence has been revealed that lots of the noncoding regions in human genome are actively transcribed.^6^, ^7^ Further sequencing and annotation of these transcripts leads to the discovery of thousands of long noncoding RNAs (lncRNAs), which is longer than 200 nt in length and lacking of protein coding capacity.^8^, ^9^ In addition, emerging studies indicate that lncRNAs are key regulators involved in almost every aspect of intracellular biological process, including the chromatin remodeling, X chromatin imprinting, cell differentiation and fate decision, immune response, tumor cells growth and metastasis etc.^10^, ^11^ Importantly, lncRNAs dysregulation have been reported in diverse human disease, especially cancers.^13^ Accumulating evidence reveals that lncRNAs exert their roles in tumorigenesis and tumor progression by repressing tumor suppressors or activating oncogenes through multiple mechanisms, including acting as cancer‐related microRNAs sponges, recruiting histone modifiers to target genes and regulating their transcription, affecting mRNA decay and translation.^14^, ^15^ For instance, Sun and colleagues reported that lncRNA HOXA11‐AS promoted gastric cancer tumorigenesis through functioning as scaffold of chromatin modification factors LSD1, PRC2, and DNMT1.^16^ In addition, lncRNA PDCD4‐AS1 contributes to breast cancer progression by stabilizing PDCD4 mRNA through forming RNA duplex and controlling PDCD4 mRNA’s interaction with RNA decay promoting factor HuR.^17^ Moreover, LINC01234 promotes the malignancy of gastric cancer via acting as a competing endogenous RNA for miR‐204‐5p to regulate CBFB expression.^18^

To date, a lot of ovarian cancer associated lncRNAs clinical relevance, biological function, and underlying mechanisms have been identified and reported. For example, overexpressed lncRNA NEAT1 is correlated with high‐grade serous ovarian cancer patients’ poor prognosis and promotes cell growth and migration through acting as a competing endogenous RNA for miR‐506.^19^ In addition, Liang et al reported that lncRNA PTAR promotes cells invasion and metastasis by sponging miR‐101‐3p to release its repression of ZEB1 in serous ovarian cancer.^20^ Moreover, LINC00092 promotes metastatic progression of ovarian cancer by binding the PFKFB2, thereby affecting glycolysis and sustaining the local supportive function of cancer‐associated fibroblasts.^21^ In this study, we performed a genome‐wide profiling analysis to identify more ovarian cancer associated lncRNAs, and explored their clinical outcome. Furthermore, we determined the oncogenic function and underlying molecular mechanism of one of the up‐regulated lncRNAs‐ LINC00511. Our findings reveal lots of novel differentially expressed lncRNAs in ovarian cancer, which may provide a valuable list of lncRNA candidates for ovarian diagnosis and treatment.

## 2 METHODS AND MATERIALS

### 2.1 lncRNA differential profiling analysis in ovarian cancer

The ovarian cancer specimen RNA sequencing data and corresponded clinical information were downloaded from https://portal.gdc.cancer.gov/ , and the normal ovarian specimen RNA sequencing data were downloaded from The Genotype‐Tissue Expression (GTEx). In addition, three public ovarian cancer microarray profiling dataset (GSE10971,^22^ GSE29450^23^, and GSE54388^24^) were downloaded from the Gene Expression Omnibus (GEO) database. For the microarray data, we applied the well‐established limma’s eBayes method to do the differential expression analysis based on the Affymetrix Human Genome U133 Plus 2.0 Array platform. For the RNASeq data, we applied the Mann‐Whiteney *U* test to the upper‐quantile normalized FPKM values of the RNASeq dataset. The *P*‐values were adjusted using the Benjamini‐Hochberg correction method. lncRNAs with log fold change (logFC) ≥1 and *P* ≤ 0.01 were defined as up‐regulated, and lncRNAs with log fold change (logFC) ≤−1 and *P* ≤ 0.01 were defined as down‐regulated.

### 2.2 Copy number amplification and deletion analysis

Each ovarian cancer specimen’s raw copy number variations data were obtained from the Broad GDAC (https://portal.gdc.cancer.gov/). Next, GISTIC 2.0^25^ pipeline was used to assess the significance of amplification or deletion of each lncRNAs’ genomic locus copy number. We aligned and mapped all lncRNAs’ genomic regions based on the location of the GISTIC peaks, and a deletion or amplification peak with a *q* value (the minimum false discovery rate at which an observed score is deemed significant) <0.25 was defined as significant. The number and focus//broad frequencies, peak *q* value of each lncRNA were integrated at the gene level.

### 2.3 Analysis of prognosis‐related lncRNA in ovarian cancer

We performed univariate Cox regression analysis using Bioconductor and R software to investigate the significance of each lncRNA in predicting overall survival (OS) in patients with ovarian cancer. Then, Multivariate Cox regression analysis was performed using Bioconductor and R software to determine if lncRNA can be used as a dependent variable factor in predicting OS in patients with ovarian cancer. Finally, patients with ovarian cancer were divided into high or low lncRNA expression groups according the lncRNA’s median expression levels, and lncRNA with log rank *P* value <0.05 between two groups was defined as significant.

### 2.4 Cell culture and transfection

The ovarian cancer cell line SKOV3 and SNU840 were purchased from The American Type Culture Collection (ATCC) and Type Culture Collection of the Chinese Academy of Sciences (Shanghai, China). SKOV3 and SNU840 cells were cultured in RPMI 1640 Medium (Invitrogen) supplemented with 10% fetal bovine serum (Invitrogen, shanghai, China), 100 U/mL streptomycin and penicillin (Invitrogen, Carlsbad, CA). All the cells were maintained in incubator at 37°C with 5% CO2. The negative control, LINC00511 and EZH2 siRNAs (Invitrogen) were transfected into SKOV3 and SNU840 cells by using RNAiMAX (Invitrogen) based on the manufacturer’s manual. Fourty‐eight hours post cell transfection, the SKOV3 and SNU840 cells were collected for RNA and total protein extraction. The LINC00511 siRNA sequences are: siRNA 1#,5′‐CCCAUGUCUGCUGUGCCUUUGUACU‐3′ siRNA 2#, 5′‐CCAGUGUGUGCUGAUGACACAUACA‐3′. The EZH2 siRNA sequence is siRNA, 5′‐AAGACUCUGAAUGCAGUUGCU‐3′.

### 2.5 RNA extraction and qRT‐PCR assays

The total RNA of SKOV3 and SNU840 cells was isolated using the RNeasy Mini Kit (QIAGEN) according to the manufacturer’s instructions. Next, 1 μg total RNA was reverse‐transcribed into cDNA by using the PrimeScript RT Kit (TaKaRa) according to the manufacturer’s instructions. For qRT‐PCR assays, SYBR Premix Ex Taq (TaKaRa) was used to detect the expression levels of LINC00511 and P21, following the manufacturer’s instructions. The house keeping gene GAPDH was used as internal control. The primer sequences of LINC00511, EZH2, P21 and GAPDH are: LINC00511, forward 5′‐CGCAAGGACCCTCTGTTAGG‐3′, reverse 5′‐GAAGGCGGATCGTCTCTCAG‐3′; EZH2, forward 5′‐TGCACATCCTGACTTCTGTG‐3′, reverse 5′‐AAGGGCATTCACCAACTCC‐3′; P21, forward 5′‐AAGTCAGTTCCTTGTGGAGCC‐3′, reverse 5′‐GGTTCTGACGGACATCCCCA‐3′; GAPDH, forward 5′‐AGAAGGCTGGGGCTCATTTG‐3′, reverse 5′‐AGGGGCCATCCACAGTCTTC‐3′.

### 2.6 Cell counting kit‐8 (CCK‐8) and colony formation assays

The proliferation ability of LINC00511 or negative control siRNA transfected cells was examined by using a CCK‐8 kit (Beyotime Institute of Biotechnology, China) based on the manufacturer’s instruction. SKOV3 and SNU840 cells transfected with LINC00511 or negative control siRNA were seeded into 96‐well plates. Ten microliters of CCK8 was added into each well, and the absorbance value at 450 nm was measured every 24 hour. For cell colony formation, LINC00511 or negative control siRNA transfected SKOV3 and SNU840 cells were seeded into six‐well plates (1000 cell/well), and incubated in incubator for 2 weeks. Next, the colonies were fixed with 4% paraformaldehyde, and stained with 0.1% crystal violet solution. The number of colonies was counted by visual inspection.

### 2.7 Subcellular fractionation

The distribution of LINC00511 in the cytoplasm and nuclear fractions of SKOV3 and SNU840 cells was determined using the PARIS Kit (Life Technologies), following the manufacturer’s protocol.

### 2.8 RNA immunoprecipitation (RIP)

The RIP assay was conducted by using the Magna RIP RNA‐Binding Protein Immunoprecipitation Kit (Millipore, USA) following the manufacturer’s protocol. The IgG and SNRNP70 antibodies were used as negative and positive control, respectively. The anti‐EZH2 (Cat# 17‐662, 5 μg/reaction) and anti‐SUZ12 (Cat# 03‐179, 5 μg/reaction) antibodies used for RIP assay were purchased from Millipore.

### 2.9 Chromatin immunoprecipitation (CHIP)

The ChIP assay was conducted by using the EZ‐ChIP Kit (Millipore, USA) followed the manufacturer’s protocol, and IgG antibody was used as negative control. EZH2 and H3 trimethyl Lys 27 antibodies (Cat# 17‐622, 5 μg/reaction) were purchased from Millipore. Specific primer of P21 promoter is as follows: forward, 5′‐GGTGTCTAGGTGCTCCAGGT‐3′; reverse, 5′‐GCACTCTCCAGGAGGACACA‐3′.

### 2.10 Statistical analysis

The Students *t*‐test (two tailed), Mann‐Whitney *U*‐test and one‐way analysis were used to analyze the in vitro data by SPSS 18.0 software. *P* < 0.05 was determined as significant.

## 3 RESULTS

### 3.1 lncRNA differential profiling analyses in ovarian cancer

**Figure 1:**
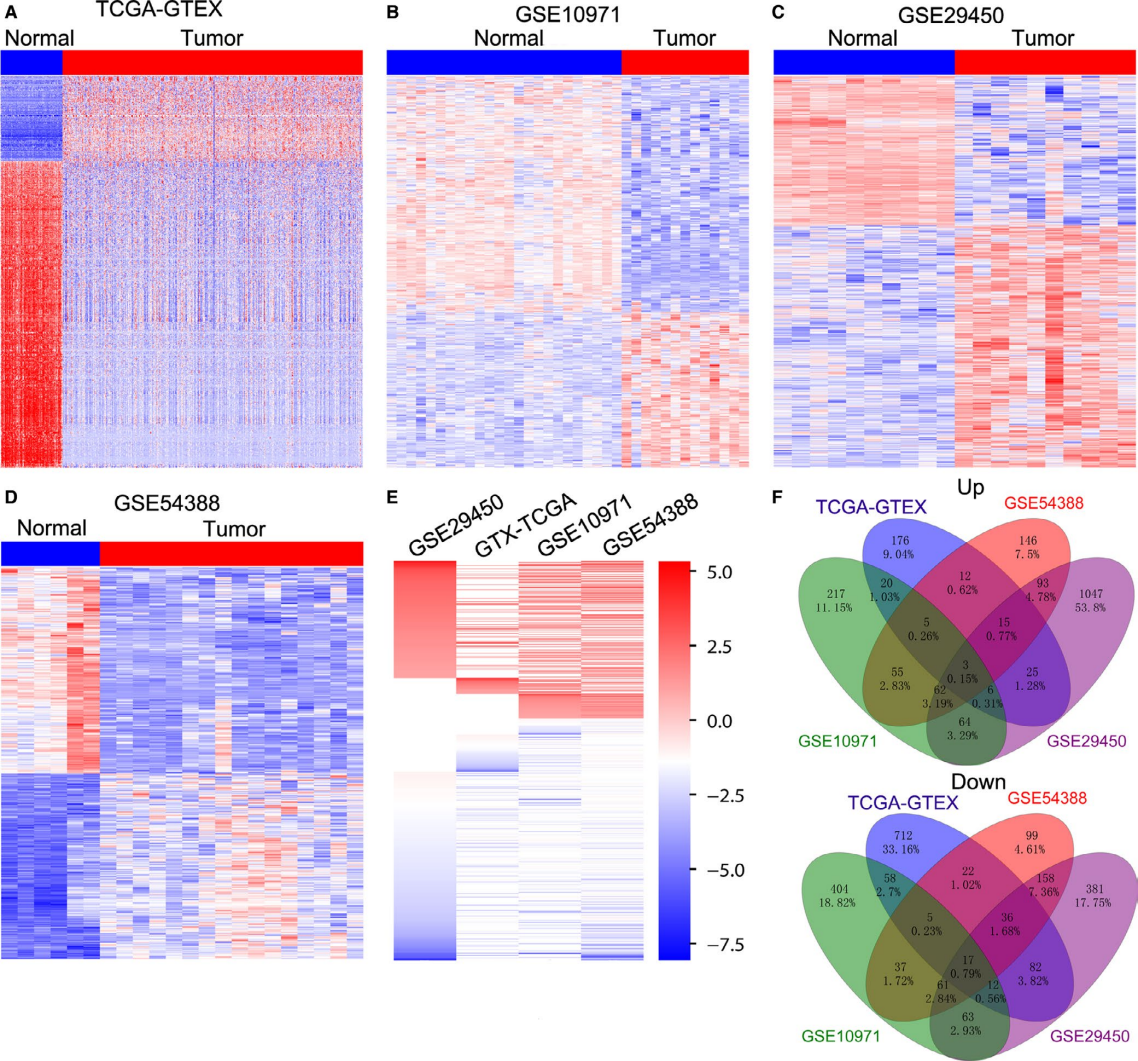
Differential profiling analyses of lncRNAs expression in ovarian cancer tissues and normal tissues. A, A heatmap was drawn to show the altered lncRNAs expression in ovarian cancer specimen from TCGA compared with normal samples from the GTEx. B‐D, Heatmaps were drawn to show the dysregulated lncRNAs in ovarian cancer specimen compared with normal tissues in the GSE10971, GSE29450, GSE54388 datasets. E, A heatmap was drawn to show the up‐regulated or down‐regulated lncRNAs (consistently in at least two datasets, fold change) in TCGA/GTEx, GSE10971, GSE29450, and GSE54388 datasets. F, Venn diagram of altered up‐regulated or down‐regulated lncRNAs in TCGA/GTEx, GSE10971, GSE29450, and GSE54388 datasets

To explore the differentially expressed lncRNAs in human ovarian cancer, we downloaded the ovarian cancer specimen RNA sequencing data from TCGA and normal ovarian samples RNA sequencing data from GTEx, and three gene microarray profiling data (GSE10971, GSE29450, GSE54388) from GEO. The TCGA data include 427 ovarian cancer samples and GTEx data consist of 88 normal ovarian samples. The GSE10971 dataset includes 13 tumor tissue samples and 24 normal control samples; GSE29450 consists of 10 ovarian cancer specimens and 10 normal ovarian surface epithelium samples; GSE54388 consists of 6 normal samples and 16 ovarian cancer specimens. Next, lncRNAs’ annotation and differential analyses revealed that 1206 lncRNAs were dysregulated in the TCGA/GTEx data (262 upregulated and 944 downregulated); 1089 lncRNAs were dysregulated in the GSE10971 dataset (432 upregulated and 657 downregulated); 2125 lncRNAs expression were altered in the GSE29450 dataset (1315 upregulated and 810 downregulated); 826 lncRNAs were dysregulated in the GSE54388 dataset (391 upregulated and 435 downregulated; Figure 1A‐D, and Table S1). Further Venn analyses revealed that 357 lncRNAs were consistently increased and 549 lncRNAs were decreased in at least two datasets，and 3 lncRNAs (FOXP4‐AS1, RP11‐283G6.4 and RP11‐7K24.3) were up‐regulated and 17 lncRNAs (TTN‐AS1, HAND2‐AS1 etc) were down‐regulated in all datasets (Figure 1E‐F， Table S2). These findings indicate that hundreds of lncRNAs’ expressions are altered in human ovarian cancer, and some of these lncRNAs can be used as novel biomarkers for ovarian cancer diagnosis.

### 3.2 Copy number variations of lncRNAS genome loci in ovarian cancer

**Figure 2:**
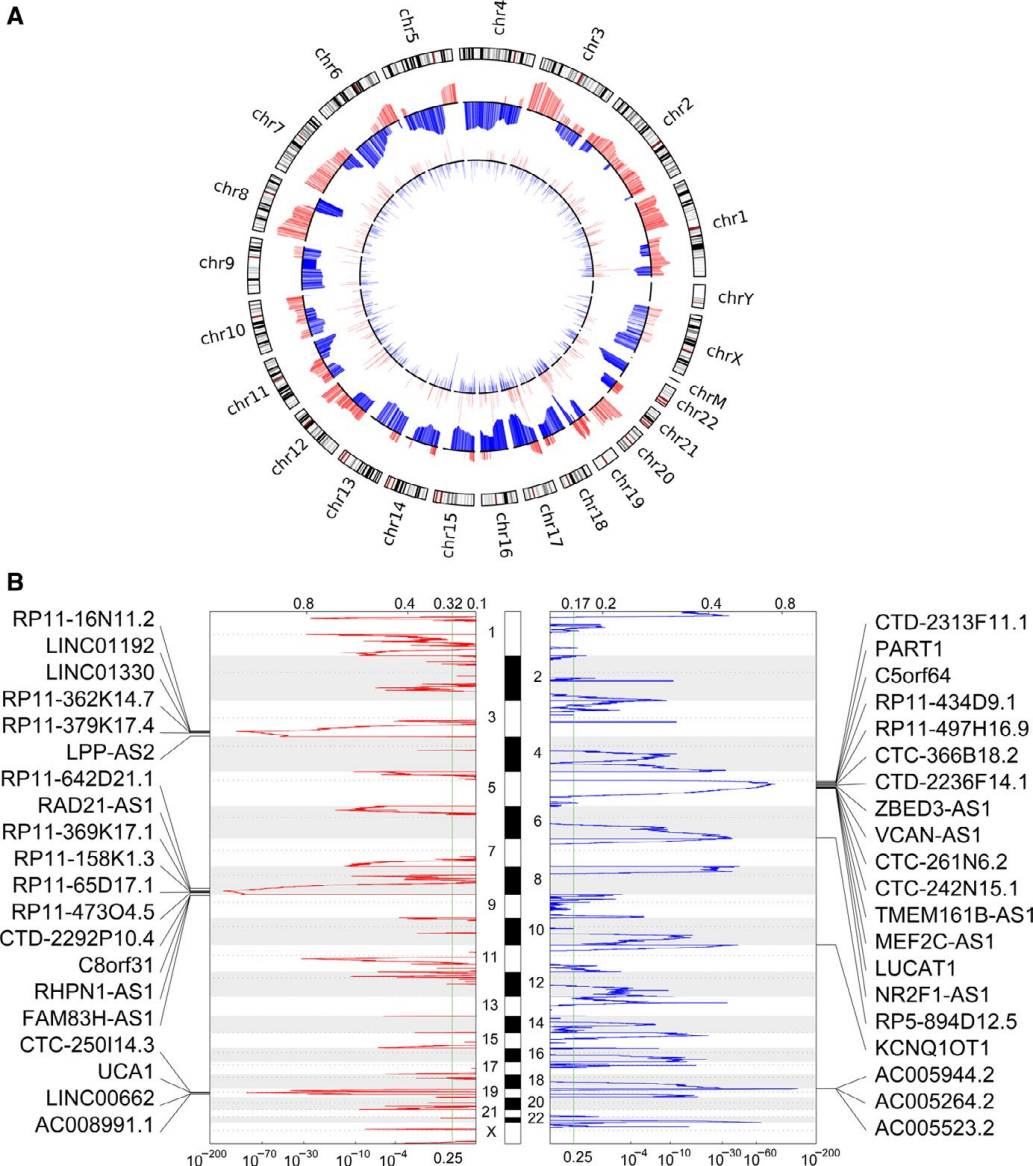
Copy number variations analyses of lncRNAs genome loci in ovarian cancer. A, A circular map was drawn to show the copy number variations of the differentially expressed lncRNAs genome loci in ovarian cancer. B, A peak map was drawn to show the frequency of up‐regulated lncRNAs copy number gain (red) in ovarian cancer tissues (top20). Each arrow represents a locus of lncRNAs, which are arranged in chromosomal order. B, A peak map was drawn to show the frequency of up‐regulated lncRNAs copy number loss (blue) in ovarian cancer tissues (top20). Each arrow represents a locus of lncRNAs, which are arranged in chromosomal order

Recent studies have shown that changes in the copy number of the genome loci may be one of the factors involving in the lncRNAs’ dysregulation in diverse tumor cells. To determine whether genome alterations contribute to lncRNAs’ dysregulation in ovarian cancer, we downloaded the ovarian cancer samples’ somatic copy number alterations data from the TCGA. Next, each differential lncRNAs’ CNVs frequencies in all ovarian cancer samples were calculated, and the alterations with *q* value <0.25 was considered as significant. Our analyses results indicate that 63/357 (~17.65%) up‐regulated lncRNAs (such as LINC01192, LINC01330 and UCA1) are accompanied by amplification of copy number, while 153/549 (~27.87%) down‐regulated lncRNAs (such as PART1, VCAN‐AS1 and NR2F1‐AS1) are accompanied by deletion of copy number (Figure 2). These findings suggest that some lncRNAs expression disorders in ovarian cancer could be related to changes in their genome somatic copy number.

### 3.3 Identifying ovarian cancer survival related lncrnas

**Figure 3:**
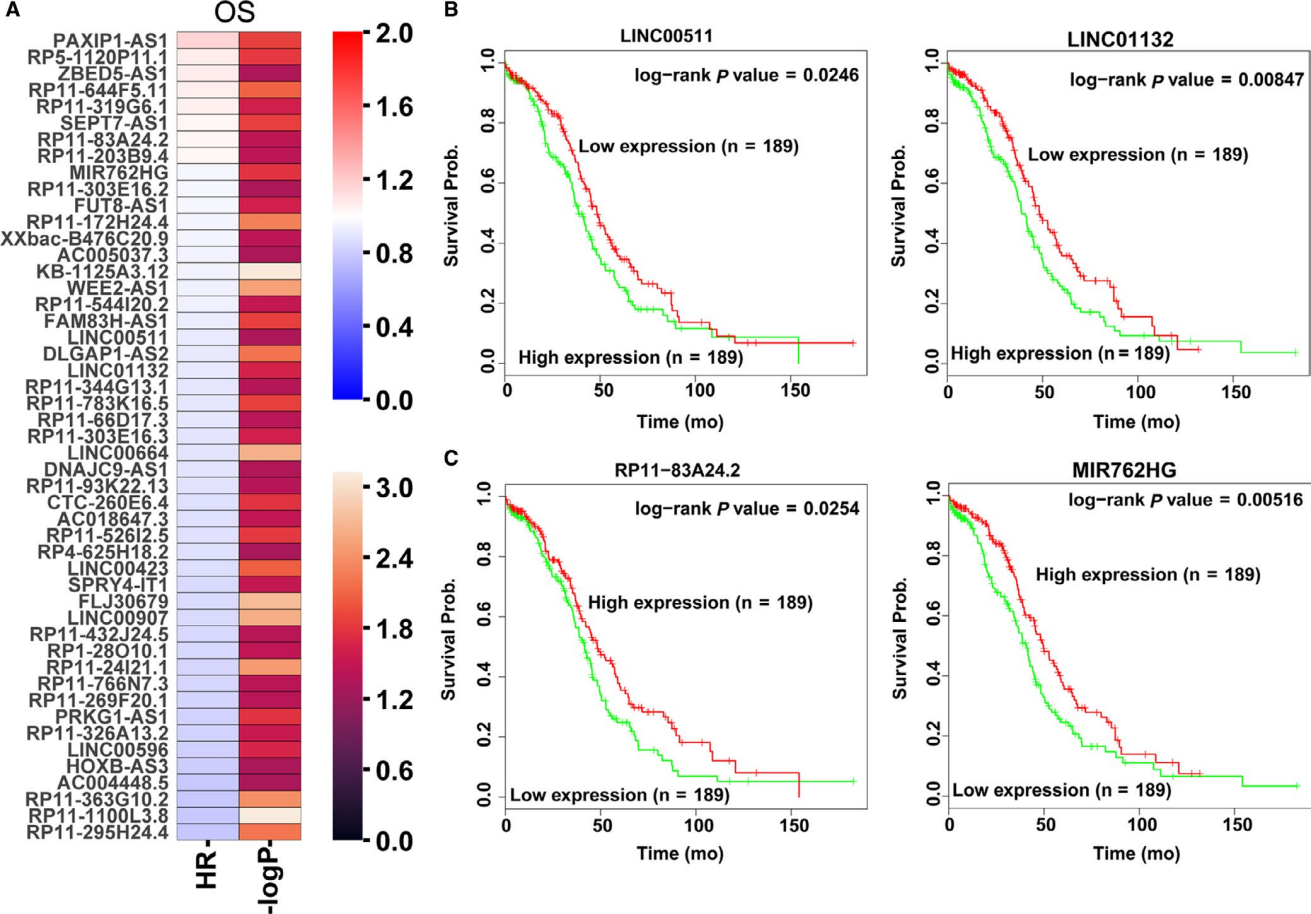
Ovarian cancer patients’ overall survival related lncRNAs analyses. A, A heatmap was drawn to show the overall survival associated lncRNAs Hazard ratio value and ‐log *P* value in ovarian cancer using the TCGA clinical data. B, The Kaplan‐Meier curve for ovarian cancer patients overall survival in high‐ or low‐ LINC00511 and LINC01132 groups using the TCGA clinical data was conducted with two‐sided log‐rank test. C, The Kaplan‐Meier curve for ovarian cancer patients overall survival in high‐ or low‐ RP11‐83A24.2 and MIR762HG groups using the TCGA clinical data was conducted with two‐sided log‐rank test

Recently, increasing number of evidence has revealed that many lncRNAs are closely related to the outcome of multiple cancer patients, and these lncRNAs can be used as independent factors in predicting the prognosis of cancer patients. To identify lncRNAs associated with prognosis in patients with ovarian cancer, we conducted univariable Cox regression analysis. As a result, we found that 22 up‐regulated lncRNAs and 25 down‐regulated lncRNAs are associated with ovarian cancer patients’ poorer OS (log rank *P* < 0.05; Figure 3A, and Table S3). Taken LINC00511, LINC01132, MIR762HG and RP11‐83A24.2 for example, ovarian cancer patients with higher LINC00511 and LINC01132 expression levels or lower MIR762HG and RP11‐83A24.2 expression levels had poorer outcome (Figure 3B and C). These data suggest that those ovarian cancer survival related lncRNAs might be useful candidates for ovarian cancer patients outcome prediction.

### 3.4 Knockdown of LINC00511 inhibits ovarian cancer cells proliferation

**Figure 4:**
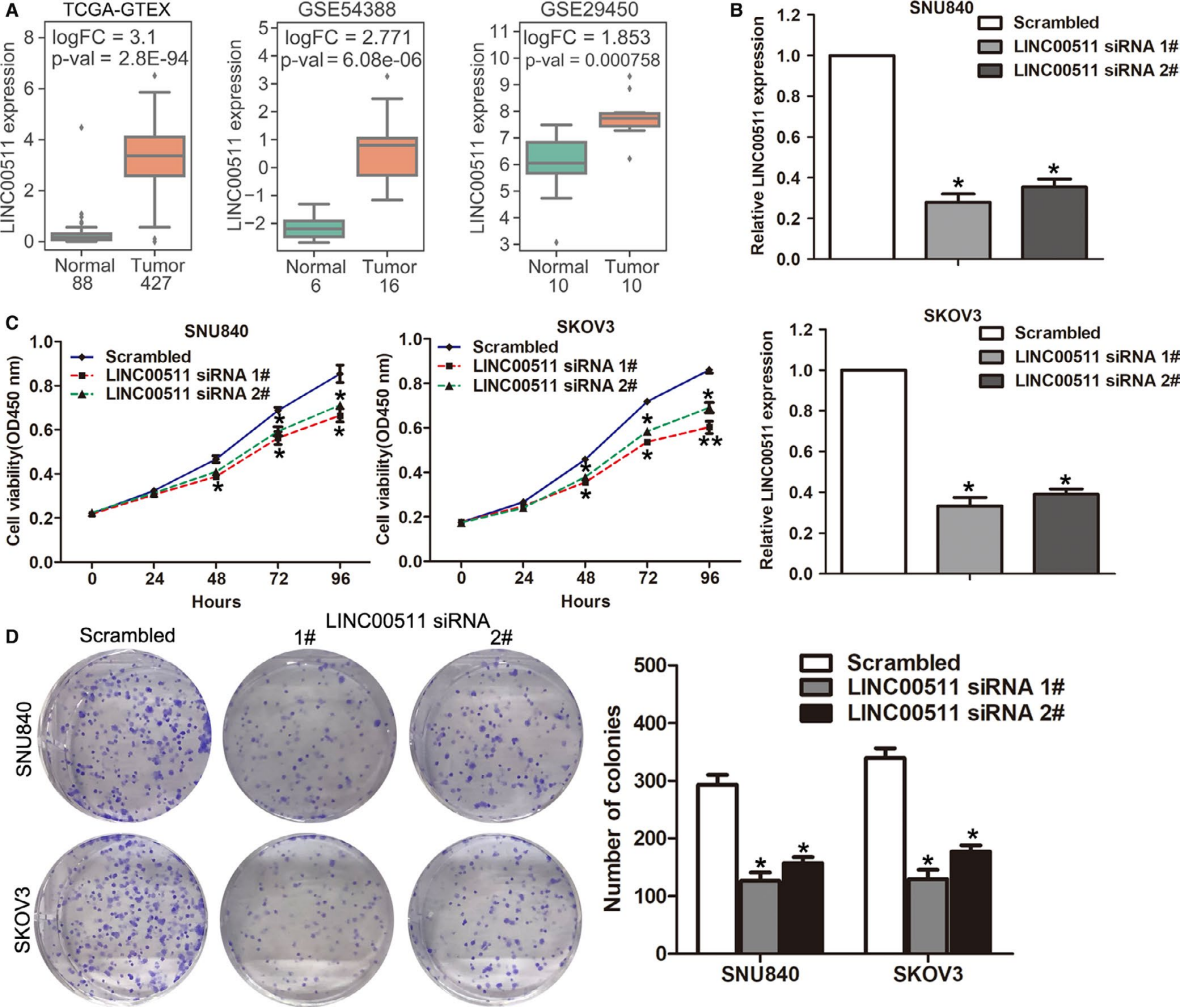
Down‐regulation of LINC00511 inhibits ovarian cancer cells proliferation. A, The relative expression levels of LINC00511 in ovarian cancer and normal tissues in TCGA/GTEx, GSE54388, and GSE29450 datasets. B, The relative expression levels of LINC00511 were examined by qRT‐PCR in SKOV3 and SNU840 cells after transfection with LINC00511 or negative control siRNAs. C, CCK8 assays was conducted to evaluate the cell proliferation ability of LINC00511 or negative control siRNA transfected SKOV3 and SNU840 cells. D, Clony formation assays were conducted to evaluate the cell colony formation ability of LINC00511 or negative control siRNA transfected SKOV3 and SNU840 cells. **P < 0.01; *P < 0.05

In order to verify whether these dysregulated lncRNAs play a biological role in ovarian cancer cells, we focus on the up‐regulated lncRNAs because these lncRNAs are more likely to act as biomarkers. Among these over‐expressed lncRNAs, LINC00511 expression is up‐regulated in TCGA/GTEx, GSE54388, and GSE29450 datasets (Figure 4A). Importantly, increased LINC00511 expression is associated with ovarian cancer patients’ poorer outcome. Hence, we chose LINC00511 as a candidate. Next, we synthesized LINC00511‐specific siRNAs and tranfected them into SNU840 and SKOV3 cells to down‐regulate its expression. The qRT‐PCR results revealed that both siRNAs can knock down the expression level of LINC00511 in SNU840 and SKOV3 cells (Figure 4B). Furthermore, CCK8 and colony formation assays revealed that down‐regulation of LINC00511 inhibited SNU840 and SKOV3 cells proliferation and colony formation ability compared with control cells (Figure 4C‐E). These results indicate that our analyses data can provide a useful list of ovarian cancer associated lncRNAs for other studies.

### 3.5 LINC00511 represses P21 expression through interacting with EZH2

**Figure 5:**
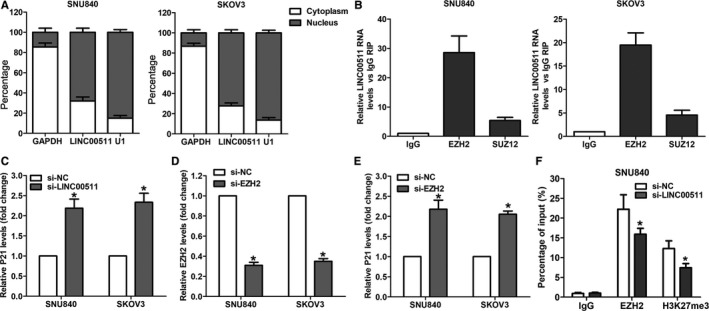
LINC00511 represses P21 expression through interacting with EZH2. A, The distribution of LINC00511 in SKOV3 and SNU840 cells cytoplasma and nucleus. B, LINC00511 RNA level in EZH2‐immunoprecipitates was detected by qRT‐PCR in SKOV3 and SNU840 cells and presented as fold enrichment compared with IgG immunoprecipitates. C‐E, qRT‐PCR analyses of P21 and EZH2 expression in SKOV3 and SNU840 cells after transfection with LINC00511, EZH2 or negative control siRNA. F, ChIP‐qRT‐PCR analyses of EZH2 and H3K27me3 occupancy in the promoter region of KLF2 in SNU840 cells after transfection with LINC00511 or negative control siRNA. *P < 0.05

To determine the underlying mechanism of LINC00511 in ovarian cancer cells, we analyzed its distribution in ovarian cancer cells. After detecting the expression level of LINC00511 in the cytoplasmic and nuclear components of ovarian cancer cells, we found that LINC00511 is mainly distributed in the nucleus of ovarian cancer cells (Figure 5A). Moreover, we performed RIP assays and found that LINC00511 directly bound with EZH2 in ovarian cancer cells (Figure 5B). Many recent studies have shown that lncRNAs could inhibit the expression levels of some factors (such as P21 and P51) that negatively regulate tumor cell proliferation by interacting with EZH2. Here, our qRT‐PCR results showed that down‐regulated LINC00511 could increase P21 expression in ovarian cancer cells (Figure 5C). Simultaneously, knockdown of EZH2 expression also up‐regulated P21 expression in ovarian cancer cells (Figure 5D and 5E). Importantly, the results of ChIP assay showed that EZH2 could bind to P21 promoter region, and knockdown of LINC00511 expression could decrease EZH2’s binding to P21 promoter region and H3K27me3 modification in ovarian cancer cell (Figure 5F). These findings indicate that LINC0511 affects ovarian cancer cell proliferation partly through binding with EZH2 and repressing P21 expression.

## 4 DISCUSSION

Benefit from the rapid development of high‐throughput techniques, a variety of cancer genomics projects have been implemented to identify genomic, epigenomic, and transcriptomic alterations and determine different molecular pathways in human cancers. These investigations have also focused on lncRNAs as well as protein‐coding genes, which have been found to be a significant regulator for a diverse of biological processes.^26^ Recently, a great number of lncRNAs in human ovarian cancers have been reported. For instance, Yan et al reported that lncRNA MLK7‐AS1 was up‐regulated in ovarian cancer tissues and cell lines, and promoted cell proliferation, migration and invasion through binding to miR‐375 and thereby reversing its inhibitory effect on regulating the Yes‐associated protein 1 (YAP1) expression in ovarian cancer.^27^ Although some ovarian cancer related lncRNAs have been identified, the functional roles of many other lncRNAs in ovarian cancer are still unknown.

In the present study, we conducted an integrated differential profiling analyses of lncRNAs expression in human ovarian cancer, and found that hundreds of lncRNAs are dysregulated in ovarian cancer tissues compared with normal tissues. Moreover, our findings revealed that genome copy number amplification or deletion is an important factor that affects lncRNAs expression in ovarian cancer. In addition, some lncRNAs closely related to the survival of patients with ovarian cancer were identified, such as LINC00511, LINC01132, MIR762HG, and RP11‐83A24.2. Among these lncRNAs, LINC00511 expression was significantly up‐regulated in ovarian cancer, and loss of function assays revealed its oncogenic role. Our findings uncover a great number of abnormally expressed lncRNAs in ovarian cancer, and may provide new insights into the etiology of ovarian cancer and useful lncRNA candidates list for further other studies.

In general, lncRNA can regulate the biological behavior of cancer cells through different molecular mechanisms, including functioning as ceRNA, interacting with RNA binding proteins, and affect the epigenetic modification of histones. In this study, we found that LINC00511 could bind with histone trimethyltransferase EZH2 in ovarian cancer cells. As well as our findings, Sun et al reported that LINC00511 could bind with EZH2 and repress P57 expression in non small cell lung cancer.^28^ Meanwhile, a growing number of studies have revealed the interaction between lncRNAs and EZH2. For example, Nie and colleagues reported that lncRNA ANRIL promotes cell proliferation in NSCLC by binding to EZH2 and recruiting it to KLF2 and P21 promoter regions to repress their transcription.^29^ In addition, Wan et al found that lncRNA PVT1 promotes NSCLC cells growth by epigenetically repressing LATS2 expression through interacting with EZH2.^30^ Interestingly, our findings also showed that knockdown of LINC00511 or EZH2 could increase P21 expression in ovarian cancer cells. Furthermore, the results of ChIP assay confirmed that EZH2 could bind to P21 promoter region accompanied by the H3K27me3 modification. These data indicate that the interaction between lncRNA and EZH2 is an important mechanism that contributes to parthenogenesis of cancer development and progression.

In summary, the findings in the present study reveal that hundreds of lncRNAs were dysregulated in human ovarian cancer tissues compared with parental normal or nontumor tissues, and most of these lncRNAs’ function are still unknown. In addition, some of these lncRNAs are associated with the outcome of ovarian cancer patients and may play key roles in ovarian cancer and progression. In the present study, we analyzed the lncRNAs profiling in human ovarian cancer and our data may provide a useful list of lncRNA candidates as prognostic biomarkers and potential targets for ovarian cancer therapy.

## CONFLICTS OF INTEREST

The authors have no actual or potential conflicts of interest to declare.
